# Phenotype-limited distributions: short-billed birds move away during times that prey bury deeply

**DOI:** 10.1098/rsos.150073

**Published:** 2015-06-17

**Authors:** Sjoerd Duijns, Jan A. van Gils, Jennifer Smart, Theunis Piersma

**Affiliations:** 1 Department of Marine Ecology, NIOZ Royal Netherlands Institute for Sea Research, PO Box 59, 1790 AB Den Burg, Texel, The Netherlands; 2 Wash Wader Ringing Group, The Old School House, Rhoon Road, Terrington St Clement, Norfolk PE34 4H, UK; 3 Chair in Global Flyway Ecology, Groningen Institute for Evolutionary Life Sciences (GELIFES), University of Groningen, PO Box 11103, 9700 CC Groningen, The Netherlands

**Keywords:** body size, distribution, food availability, *Limosa lapponica*, morphology, resource use

## Abstract

In our seasonal world, animals face a variety of environmental conditions in the course of the year. To cope with such seasonality, animals may be phenotypically flexible, but some phenotypic traits are fixed. If fixed phenotypic traits are functionally linked to resource use, then animals should redistribute in response to seasonally changing resources, leading to a ‘phenotype-limited’ distribution. Here, we examine this possibility for a shorebird, the bar-tailed godwit (*Limosa lapponica*; a long-billed and sexually dimorphic shorebird), that has to reach buried prey with a probing bill of fixed length. The main prey of female bar-tailed godwits is buried deeper in winter than in summer. Using sightings of individually marked females, we found that in winter only longer-billed individuals remained in the Dutch Wadden Sea, while the shorter-billed individuals moved away to an estuary with a more benign climate such as the Wash. Although longer-billed individuals have the widest range of options in winter and could therefore be selected for, counterselection may occur during the breeding season on the tundra, where surface-living prey may be captured more easily with shorter bills. Phenotype-limited distributions could be a widespread phenomenon and, when associated with assortative migration and mating, it may act as a precursor of phenotypic evolution.

## 1. Introduction

Most organisms on the Earth live in seasonal environments with respect to climate and food [1]. The ability of individuals to reversibly change phenotype in response to a change in environmental conditions is called phenotypic flexibility [2,3]. Animals making adjustments in digestive organ size to cope with different prey types or prey quality represent a well-known example of (often seasonally structured) phenotypic flexibility (e.g. [4–6]). However, some aspects of the phenotype are essentially inflexible. Traits such as bill length in birds that show determined growth are hardly flexible [7,8]. Bill morphology is a strong predictor of foraging niche (e.g. [9–15]), and may lead to phenotype-related differences in diet [16,17]. In addition, in response to environmental change, animals can show behavioural responses such as changes in foraging time (e.g. [18,19]), diet [20,21], or the movement to sites where good food may be more favourable (e.g. [22–24]).

Intra-population variation in dietary optima, and temporal and spatial variation in the abundance or availability of different prey is known for many species of fish, amphibians, insects, mammals and birds [25]. Body size, dominance, prior residency or food availability appear to be responsible for individual differences in migratory tendencies within populations [26]. Food availability is relatively easy to quantify in intertidal areas [27], and non-breeding shorebirds provide a good system for correlating distribution of animals with their food resources (e.g. [28–30]). Non-breeding shorebirds in temperate zones mostly feed on benthic prey that tends to bury deeper in winter than in summer (e.g. [31,32]). Although burying depth may be ultimately determined by climatic factors, the seasonal rhythm of burying depth for a certain location appears a response to changes in day length rather than changes in seawater temperature, at least in the case of polychaetes [32]. With seasonally changing fractions of benthic prey burying beyond the bill lengths of most shorebird species (e.g. [33,34]), the part of the population for which too high a proportion of prey has become inaccessible should move elsewhere. This could lead to ‘phenotype-limited’ forager distributions, a term that was first used to predict spatial distributions of individuals differing in dominance [35].

Bar-tailed godwits (*Limosa lapponica*) are sexually dimorphic migratory shorebirds, with females having 25% longer bills than males and mainly feeding on deep burying lugworms (a polychaete worm, *Arenicola marina*), while the shorter-billed males mainly forage on shallow-buried prey [36,37]. Among the available benthic prey items, seasonal variation in burying depth is largest in lugworms [32], so the potential for a phenotype-limited distribution should be most pronounced in female godwits. In addition, there is considerable variation in bill length within the sexes [38–40]. Although the larger sex (females) should incur lower energetic costs per unit body mass, the differential distribution between the sexes is best explained by sex-specific prey availability [41]. We therefore hypothesized that this differential distribution could be extended to individuals within a sex and tested whether phenotype-limited distributions in female bar-tailed godwits exist. Individuals with shorter bills are predicted [42] to (i) move to more favourable wintering sites (i.e. areas with prey buried less deeply) and/or (ii) switch to prey items that are buried less deeply to sustain their minimum intake requirement.

**Figure 1.**
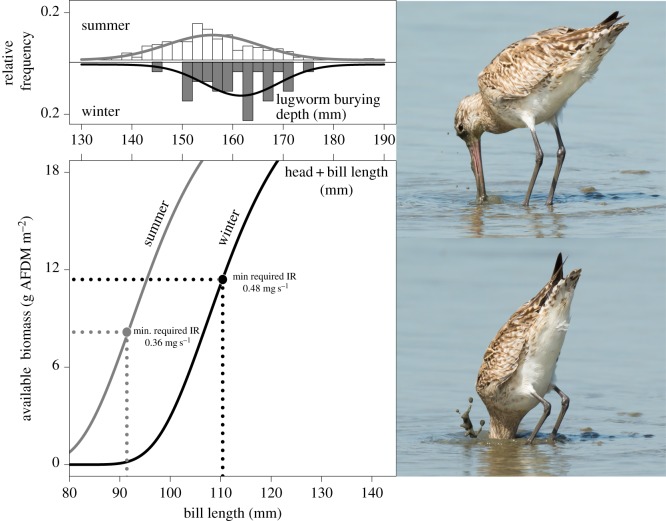
Conceptual model of available lugworm biomass in relation to bill length of female bar-tailed godwits in the Dutch Wadden Sea. Upper panel shows the distribution of individual lugworm burying depths, measured in 1981/1982 (corrected for accessibility; i.e. 40 mm subtracted), and separated for summer (April–September) and winter (October–March). From this, the availability in relation to bill length can be deduced (lower panel, in which upper axis expresses head + bill). In summer, available biomass increases steeply as a function of bill length (due to the shallower burying depth), while minimum required intake rate (IR; 0.36 mg AFDM s^−1^) is relatively low. Therefore, almost all individuals (more than 92 mm bill) are able to reach their minimum requirement foraging only on lugworms. In winter, available biomass only increases at longer bills and, furthermore, minimum requirement is relatively high (due to higher maintenance costs [43]). Shorter-billed females cannot acquire their minimum requirement and are predicted to leave this wintering site or shift their diet towards more accessible prey. Minimum requirements were calculated as follows: minimum requirement=(DEE_season_/*e*)/*T*_*f*_, where *e* is a lugworm's energy content (22 kJ g^−1^ AFDM) [32], the required daily energy expenditure DEE per season was set at 2.4×BMR in winter and 1.8×BMR in summer [43], daily foraging time *T*_*f*_ was assumed to be 12 h for both seasons (i.e. 50% [43]), assuming an assimilation efficiency of 80% [43]. The photos on the right exemplify the ability of female bar-tailed godwits to reach depths beyond the bill length. Original photos by Dave Montreuil.

Females with longer bills would be able to reach a larger fraction of the available biomass compared with shorter-billed individuals. This idea is shown in figure 1. We explored the possibility of a phenotype-limited distribution by analysing the monthly distribution in bill lengths using long-term datasets of measured and marked non-breeding females in the Dutch Wadden Sea and in the Wash, UK. To estimate how intake rates depend on prey burying depth, and to predict the observed seasonal changes in diet composition [37], we used generally applicable functional response parameters [44].

## 2. Material and methods

### 2.1 Study species

The bar-tailed godwit is a sexually dimorphic long-distance migratory shorebird, of which two subspecies are identified along the East-Atlantic flyway [45]. The subspecies *L. l.* *taymyrensis* mainly winters in West Africa, breeds in northern Siberia and uses the Wadden Sea area twice a year as a refuelling site. The *L. l.* *lapponica* subspecies winters in Northwestern Europe and breeds in northern Scandinavia [40,46]. To explore the possibility of a phenotype-limited distribution, we initially distinguished between the subspecies, as the *taymyrensis* subspecies has on average a shorter bill length than the nominate *lapponica* subspecies [40,45], with considerable overlap in morphometrics. The subspecies occur together in the Dutch Wadden Sea during six months of the year (April–October) [40]. During this period, they would encounter similar environmental conditions in the Dutch Wadden Sea, and therefore all females with known bill lengths from known and unknown sub-specific identity were included in the analyses.

### 2.2 Sightings and catches of marked individuals

Birds were caught with ‘wilsternets’ [47] or mist nets at various locations throughout the Dutch Wadden Sea area. Before release, length of bill (exposed culmen, from tip of bill to base of feathers), wing (flattened and straightened), tarsus and mass were measured using standard methods [48]. Captures (*n*=2433) and sightings of marked individual females in the Dutch Wadden Sea (*n*=4069) were analysed over the period from capture up to May 2014 to assess bill length distributions per month. The 4069 sightings were based on 1541 individuals, of which 864 individuals were sighted multiple times (i.e. different months and/or years). They were all included in the analysis, as the analyses with and without multiple sightings did not differ, while the repeated presence of an individual is considered indicative of a preference to reside at a site. Full details on number of birds caught and sighted per month and year are given in table 1. To compare bill length distributions with another major non-breeding site, biometric data were obtained from the Wash Wader Ringing Group in the UK. Here, bar-tailed godwits have been caught on the Wash with both cannon nets and mist nets [49]. The data for 1693 female bar-tailed godwits were collected in 1994–2011.

**Table 1.** Overview of numbers of female bar-tailed godwits caught and sighted in the Dutch Wadden Sea, by year and by month. Note that individuals may be sighted more than once in the same month.

year	no. caught	no. sightings	month	no. caught	no. sightings
2001	94	8	Jan	0	9
2002	99	10	Feb	9	28
2003	287	162	Mar	18	122
2004	149	146	Apr	117	237
2005	126	206	May	2057	2504
2006	180	276	June	0	38
2007	133	379	July	39	190
2008	79	172	Aug	90	641
2009	211	360	Sep	60	234
2010	262	425	Oct	38	42
2011	332	658	Nov	5	10
2012	257	588	Dec	0	14
2013	224	486			
2014	0	193			

Although seasonal differences in bill length distributions have been reported in several bird species, differential bill wear was held responsible for this variation (e.g. [50–52]). For shorebirds, it is known that the rhamphotheca, the horny covering of a bird's bill, constantly grows at the base of the bill. Despite this growth, the bill wears and within individual variation appears to be negligible (less than 1 mm; [7]). Indeed, recaptures (more than 1 year interval) of marked bar-tailed godwits show no evidence of intra-individual variation in bill length (*F*_1,12_=936.5, *R*^2^=0.99, *p*<0.001; slope=0.95 s.e. 0.03 and intercept=4.5 s.e. 2.6).

### 2.3 Prey availability

The burying depth, density and length of lugworms was measured each month in the eastern part of the Dutch Wadden Sea along the mainland coast of the province of Friesland (53°25′ N, 6°04′ E) during two consecutive years (1980/1981) [32]; the principal investigator (L. Zwarts) ensured that the original raw data became available for later analysis. Burying depth was measured as the distance between the surface and the deepest point of their U-shaped burrow [32]. As lugworms will be captured as their tail resides in one of their vertical shafts, while their body is in the bottom of the U-shaped burrow [53], we subtracted 40 mm (i.e. half of the mean length of lugworms; *n*=205) from each depth measurement, to represent availability.

### 2.4 Predicting intake rates

To examine whether the predicted energy intake rate (PEIR) was related to lugworm burying depth, we averaged monthly prey burying depths (*n*=205) and predicted intake rates throughout the year based on functional response parameters [44]. Note that the bill lengths of the birds used in the published experiment (91.4, 93.7, 94.6, 98.5 and 99.5 mm, respectively) coincided with population averages (mean=96.2±0.06 s.e, *n*=2433), and no effects of bill length were detected. For these reasons, PEIR should fairly represent population averages. By using the slope and intercept of a linear model of the searching efficiency on prey burying depth [44], we here estimated depth *i* specific searching efficiency *a*_*i*_. Searching efficiency was independent of prey length and density [44]. Handling time *T*_*h*_ was independent of prey burying depth and constant for prey density but increased with prey length [44]. Therefore, we here used the intercept and slope from a linear model of handling time against prey length to estimate length-specific handling times [44]. The month-specific predicted energy intake rate (PEIR_*m*_) was calculated using the following equation:

2.1 $${\mathrm{PEIR}}_{m}=\sum_{ij}^{ij}\frac{\left(a_{i}N_{ijm}e_{j}\right)}{\left(1+a_{i}N_{ijm}T_{hj}\right)},$$

where *N* is the mean density (# m^−2^) for depth *i*, prey length *j* and month *m*, as measured by Zwarts & Wanink [32], and *e* the ash-free dry mass (AFDM (mg); i.e. energetic value) per individual prey using the length–AFDM relation (e.g. [36,54]). Next, we evaluated the mean monthly energetic contribution of lugworms to the year-round diet based on field observations (*n*=76) [36,37,55] and dropping analyses (*n*=240) [54].

### 2.5 Statistical analyses

Monthly lugworm burying depth and monthly bill length distributions (with and without subspecies differentiation) were explored using linear and quadratic models. In the linear (null) model, prey burying depth or bill length did not depend on month. The alternative (quadratic) model was evaluated using model selection methods and ranked using Akaike Information Criterion (AIC), and the model was considered to be substantially better when the AIC value was at least two points lower when compared with the other model [56]. To assess the proportion of available prey in relation to bill length, the empirical cumulative distribution function (ECDF [57]) was plotted for females captured or sighted in summer (April–September) and winter (October–March). All analyses were conducted using R v. 3.0.1 [58].

## 3. Results

**Figure 2.**
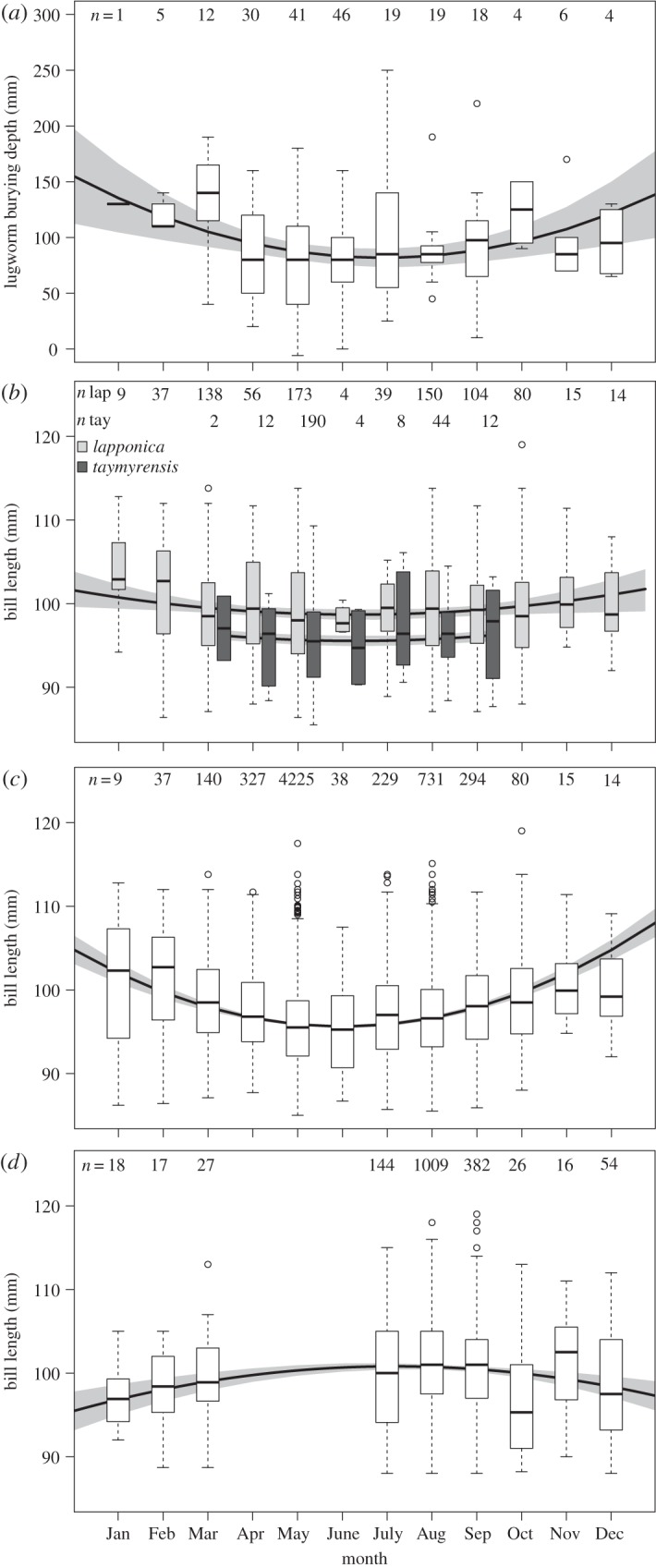
By month, (*a*) lugworm burying depth in the Dutch Wadden Sea, (*b*) bill length distributions of female bar-tailed godwits in the Dutch Wadden Sea, separated for both subspecies, (*c*) as (*b*) but now subspecies pooled and (*d*) bill length distributions for female bar-tailed godwits in the Wash, subspecies pooled. Curved lines represent model outputs and grey areas represent 95% confidence intervals (CIs). Box plots show median (line in box), interquartile range (box), 10th and 90th percentiles (bars) and outliers (dots). Sample sizes are shown in all plots.

**Figure 3.**
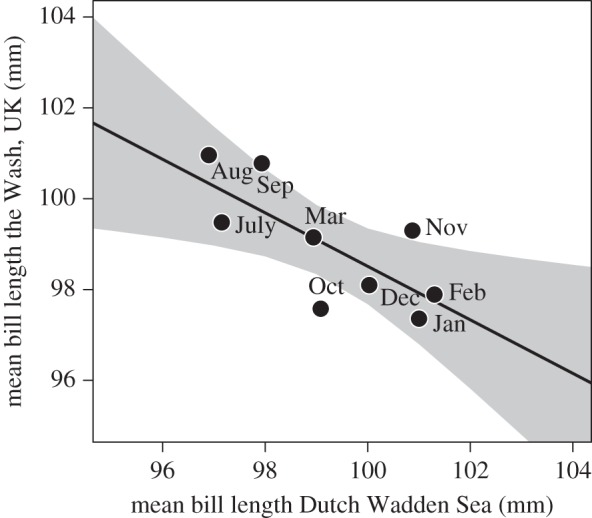
Correlation between monthly mean bill lengths of bar-tailed godwits captured in the Wash and resighted in the Dutch Wadden Sea. Grey shading represents 95% CI level.

Burying depth of lugworms varied predictably throughout the year (figure 2*a*). Lugworms bury deepest during winter (*F*_2,202_=5.03, *R*^2^=0.05, *p*=0.007). The bill length distribution of both subspecies showed comparable seasonal trends (*F*_3,1087_=28.06, *R*^2^=0.07, *p*<0.001; figure 2*b*). The mean bill length of the *lapponica* subspecies showed a decrease in length from January towards spring and summer, whereas from August onwards bill length increased again. As expected, bill lengths of *taymyrensis* females were shorter than of *lapponica* (*p*<0.001), though this subspecies showed the same pattern during the seven months they occurred in the Dutch Wadden Sea (figure 2*b*). Not surprisingly then, the bill length distribution of all sighted individuals with known bill lengths showed a strong seasonal trend (*F*_2,6105_=95.45, *R*^2^=0.03, *p*<0.001; figure 2*c*). That shorter-billed females may have moved from the Dutch Wadden Sea towards the Wash was indicated by the inverse relationship of monthly bill length distributions in the course of the non-breeding season (*F*_2,1690_=11.81, *R*^2^=0.013, *p*<0.001 figure 2*d*). The disappearance of the shorter-billed females from the Dutch Wadden Sea was also indicated by the negative relationship between the mean bill lengths of the Wash and the Dutch Wadden Sea (*F*_1,7_=8.53, *R*^2^=0.49, *p*=0.020; figure 3).

**Figure 4.**
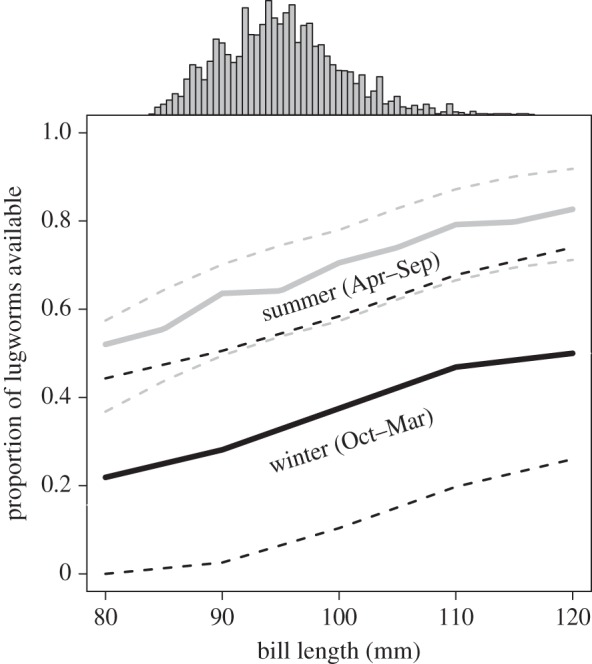
Summer and winter pattern of the (numerical) proportion of accessible lugworms in relation to bill length, based on the year-round depth measurements of lugworms in the Dutch Wadden Sea. Solid lines represent mean summer (grey) and mean winter (black) lugworm availability (dashed lines represent the 95% CI levels). In either season availability increases with an increase in bill length, though in winter overall availability is much lower. Bars on top of the graph denote the frequency distribution of bill length of female bar-tailed godwits captured in the Wadden Sea (*n*=2433).

**Figure 5.**
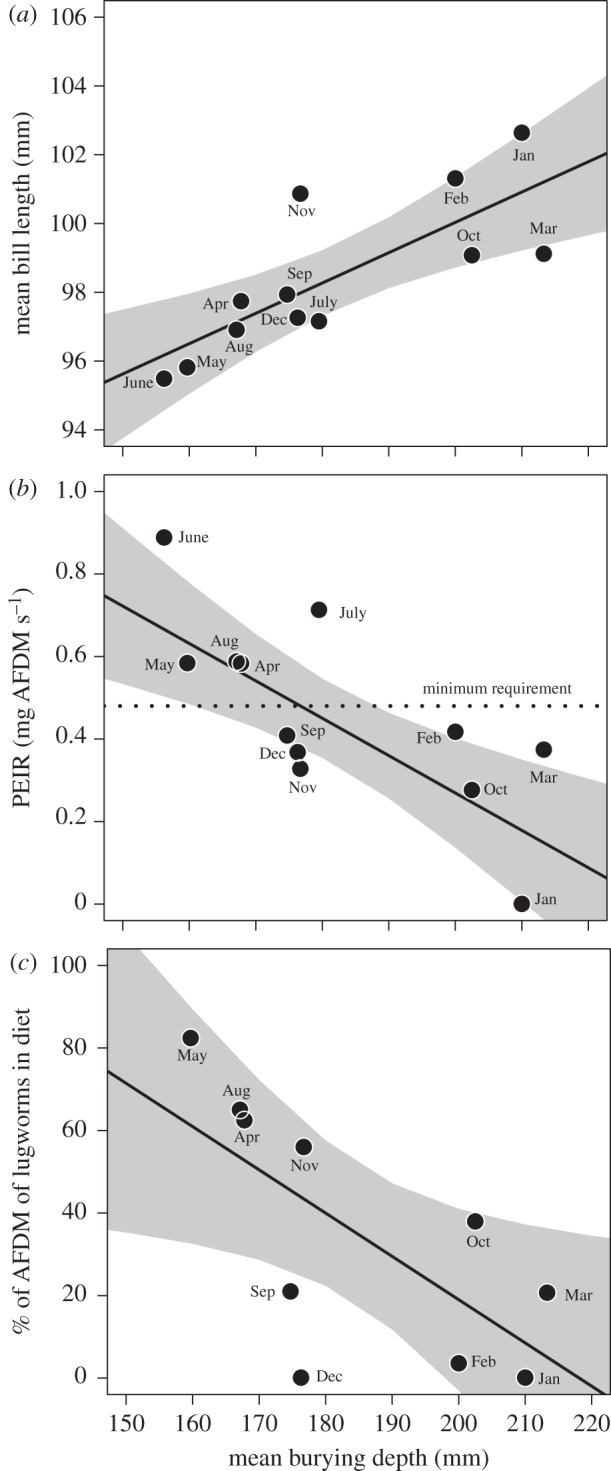
Effect of prey burying depth on bill length, intake rate and diet composition of bar-tailed godwits present in the Dutch Wadden Sea. (*a*) Mean bill lengths of females are larger in winter, when prey are buried deeper (the grey area represent the 95% CI level). (*b*) PEIRs, based on parameters from the functional response, correlate negatively with mean burying depth. (*c*) The mean contribution of lugworms to the diet of female bar-tailed godwits (based on AFDM) increases when lugworms are closer to the surface.

In winter, a longer bill is needed to access the same proportion of prey available as in summer (figure 4), which is the likely explanation for the positive correlation between mean monthly burying depth and mean bill length (*F*_1,10_=15.20, *R*^2^=0.60, *p*=0.003; figure 5*a*). There was a clear negative correlation between burying depth of lugworms and predicted intake rate (PEIR), suggesting that in winter some bar-tailed godwits would not be able to satisfy their minimum energy requirement by foraging on lugworms only (*F*_1,10_=12.24, *R*^2^=0.55, *p*=0.006; figure 5*b*). Indeed, individuals remaining in the Dutch Wadden Sea in winter included prey other than lugworms in their diet; the energetic contribution (% of AFDM) of lugworms was negatively correlated with lugworm burying depth (*F*_1,8_ = 6.97, *R*^2^=0.40, *p*=0.030; figure 5*c*).

## 4. Discussion

The vast majority of studies of changing resource landscapes and their use by animals has focused on differences between species or sexes (e.g. [16,59–61]). However, it is the variation between individuals that provides the raw material for evolutionary and ecological processes [62–64]. Here, we provide an example of a seasonally changing phenotype-limited distribution in one species and in one sex.

Female bar-tailed godwits redistributed in accordance with the seasonal changes in availability of their dominant prey. In winter, when lugworms are buried more deeply, individually marked shorter-billed individuals were no longer seen in the Dutch Wadden Sea. One of the areas they moved to is probably the Wash, where during the winter months an increase in numbers has been observed [65]. Indeed, while shorter-billed individuals disappeared from the Wadden Sea during winter, there was a build-up of such individuals in the Wash. This pattern is consistent with the finding that in the climatically more benign Wash, benthic prey are buried less deeply than in the Dutch Wadden Sea [41].

The lugworm data were collected long before most of the data on bar-tailed godwits, and mean seawater temperature increased over the last three decades by about 1.2°C [66]. This increase in average seawater temperature is unlikely to have biased our lugworm availability assessment because, in the short term, burying depth appears unrelated to temperature, i.e. it has no effect on depth within months [32].

In addition to the increased burying depth of lugworms in the colder winter months, there is an additional reason why lugworms will be more difficult to capture in these months. Bar-tailed godwits rely on cast-formation (defaecation) to detect lugworms and in the colder and thus metabolically more inactive winter months they produce fewer casts [34,53]. Together these two factors could result in bar-tailed godwits failing to achieve their daily required intake when only eating lugworms. Therefore, even the longer-billed individuals are predicted to add smaller, less profitable prey species to their diet. That a more varied diet was indeed observed in various places across coastal Europe [37,54] emphasizes once more that sex-specific food availability is a main driver of winter distributions [64].

If the intake rate benefits accrued by longer-billed individuals result in long-term fitness benefits, there should be directional selection for a longer bill. However, bar-tailed godwits breed on the tundra where they feed mainly on surface and shallow-buried arthropods, also available to their shorter-billed self-foraging chicks [67,68]. It has been suggested that shorter bill sizes may actually be advantageous when feeding on such prey [69,70], suggesting that there may be selection for longer bills in winter and shorter bills in summer.

After unpredictable extreme conditions such as prolonged drought or cold spells, some phenotypes with particular body size values may die, while other phenotypes survive or even benefit from these events. The available examples (e.g. [71–73]), however, pertain to resident or territorial birds that do not move away. In most birds however, individuals often move, and as most benthic prey show seasonal variations in burying depth [32], phenotype-limited distributions are likely to be found more in species dependent on benthic prey. In fact, we predict that phenotype-limited distributions occur across a range of taxa with reference to a range of traits.

## 5. Conclusion

By examining a fixed aspect of the phenotype (in our case bill length), we could show that female bar-tailed godwits redistribute themselves across soft-sediment systems along the southern North Sea coast in accordance with the seasonal changes in the availability of their dominant prey. Phenotype-limited distributions could be widespread and, when associated with assortative migration and mating, they may act as precursors of phenotypic evolution.

## Data accessibility

Data used in this study are deposited in Dryad: http://dx.doi.org/doi:10.5061/dryad.v51t0.
